# Optimization of dsRNA Length and Target Position for Enhanced RNA Interference Efficiency Targeting the Chitinase Gene *MsChi2* in *Mythimna separata*

**DOI:** 10.3390/insects17090931

**Published:** 2026-09-04

**Authors:** Wenhua Bao, Yan Qiu, Xiaoke Zhao, Danyang Sun, Feng Gao

**Affiliations:** 1 College of Life Science and Technology, Inner Mongolia Normal University, Hohhot 010022, China; 20220047@imnu.edu.cn (W.B.); 20254015014@mails.imnu.edu.cn (Y.Q.); 20254015057@mails.imnu.edu.cn (X.Z.); 20244015046@mails.imnu.edu.cn (D.S.); 2 Key Laboratory of Biodiversity Conservation and Sustainable Utilization in Mongolian Plateau for College and University of Inner Mongolia Autonomous Region, Hohhot 010022, China

**Keywords:** *Mythimna separata*, chitinase, RNA interference, dsRNA length, target position, pest control

## Abstract

Chitinase is a key enzyme involved in insect chitin metabolism, and RNA interference (RNAi) targeting the corresponding gene represents a promising strategy for eco-friendly pest management. However, the design parameters of double-stranded RNA (dsRNA), particularly fragment length and target position, remain insufficiently optimized. To investigate the effects of these parameters on the RNAi efficiency against the *MsChi2* gene in *Mythimna separata*, we synthesized four dsRNAs of 60, 200, 500, and 1000 bp and administered them to larvae. The results indicated that 200 and 500 bp dsRNAs significantly inhibited larval growth, reduced survival, and silenced *MsChi2* expression. Among these, the 200 bp dsRNA exhibited the most pronounced effects, including a 59% inhibition rate of larval body weight, 65.9% mortality, and 62% target gene silencing efficiency, accompanied by a significant reduction in chitin content and abnormalities in molting and pupation. Using 200 bp as the optimal length, five fragments targeting different coding regions of the gene were evaluated. All except one exhibited strong activity, and their RNAi efficiencies were positively correlated with the predicted numbers of effective small interfering RNAs. Collectively, these findings demonstrate that dsRNA length and target position jointly modulate RNAi efficiency, with 200 bp representing the optimal fragment length. This study provides a rational design framework for dsRNA development and establishes both theoretical and practical foundations for RNAi-based management of *M. separata*.

## Simple Summary

*Mythimna separata* is a destructive pest of major cereal crops, including maize, wheat and rice, causing severe yield losses worldwide. Current management relies heavily on chemical insecticides, which pose risks to the environment and lead to resistance. RNA interference is a biological approach that uses double-stranded RNA molecules to switch off essential genes in insects. In this study, we investigated how the length of these RNA molecules and target site selection within the gene affect their ability to control this pest. Our results revealed that a 200 bp RNA fragment was most effective, resulting in high mortality and significantly reducing larval growth. We also found that targeting specific regions of the gene produced greater gene-silencing efficacy than targeting other regions. These findings provide practical guidance for designing more effective RNA-based insecticides, supporting the development of environmentally friendly pest management strategies for sustainable agriculture.

## 1. Introduction

The oriental armyworm, *Mythimna separata* (Walker) (Lepidoptera: Noctuidae), is a major polyphagous migratory pest widely distributed across the principal agricultural regions of Asia and Oceania [1]. Owing to its broad host range, high reproductive capacity, and severe outbreak potential, this species causes substantial yield losses in major cereal crops such as maize, wheat, and rice every year, posing a serious threat to agricultural production and food security [2,3]. Current management of *M. separata* relies predominantly on chemical insecticides such as chlorantraniliprole. However, the long-term and extensive use of chemical pesticides has accelerated the development of insecticide resistance and resulted in significant adverse environmental effects [4]. Consequently, developing novel environmentally friendly pest control technologies with precise target specificity has become an urgent challenge in the field of plant protection.

RNA interference (RNAi) is a post-transcriptional gene silencing mechanism triggered by double-stranded RNA (dsRNA) [5,6]. In the RNAi pathway, exogenously introduced dsRNA is cleaved by the Dicer enzyme into 21–24 nt small interfering RNAs (siRNAs). These siRNAs are then incorporated into the RNA-induced silencing complex, where they guide the recognition and degradation of cognate messenger RNAs. This process enables the specific knockdown of target genes at the post-transcriptional level, thereby disrupting normal growth and development in pests and ultimately causing death [5,7].

Chitin is a major structural component of the insect cuticle, tracheae, and peritrophic membrane and is essential for molting, metamorphosis, and morphogenesis [8]. Chitinase is responsible for degrading chitin in the old cuticle during insect molting and represents a key class of enzymes regulating growth and development [9]. In lepidopteran insects, most chitinase genes belong to the glycoside hydrolase family 18 (GH18) and generally exhibit a multi-domain architecture comprising a signal peptide, a chitin-binding domain, and a Glyco_18 catalytic domain [10]. RNAi-mediated silencing of chitinase genes induces molting defects, pupal malformation, and increased mortality in insects, as validated in silkworm and cotton bollworm [11]. Because chitin and chitinases are absent from humans and vertebrates, targeting them fulfills an important prerequisite for vertebrate biosafety in RNAi-based pest control. However, the overall specificity and environmental safety of a dsRNA-based product depend not only on the absence of the target in vertebrates, but also on the sequence complementarity to potential off-target transcripts in non-target arthropods. Therefore, although *MsChi2* is a promising target, potential sequence-dependent off-target effects on beneficial or non-target organisms should be carefully evaluated and minimized during dsRNA design, for instance, by comprehensive homology screening against the transcriptomes of non-target species. Although previous studies have confirmed the feasibility of RNAi targeting *MsChi2* using various delivery systems [12,13,14], critical dsRNA design parameters, including fragment length and target site selection, have not been systematically optimized. Consequently, the present study aimed to determine the optimal dsRNA design parameters for *MsChi2* to maximize RNAi efficiency.

However, RNAi efficiency in lepidopteran pests is constrained by multiple factors, among which dsRNA length is a key determinant. However, no universally optimal length has been established; consequently, empirical optimization is required for each specific target gene and pest species [7]. Long dsRNAs (>60 nt) are generally more effective than short RNAs (<27 nt). This difference is associated with midgut uptake efficiency and Dicer-mediated processing [15,16]. However, the relationship between dsRNA length and RNAi efficiency is not simply linear. For instance, in *Leptinotarsa decemlineata*, a 200 bp fragment targeting the *β-actin* gene was more effective than a 297 bp fragment when expressed in a chloroplast plastid-based system. Conversely, a 670 bp fragment targeting the *ACE1* gene outperformed an 870 bp fragment. These findings suggest that an optimal dsRNA length is maximized within an optimal range of dsRNA lengths rather than increasing proportionally with fragment size [15,17]. Mechanistically, differential degradation of dsRNAs of varying lengths by hemolymph nucleases may explain the nonlinear changes in RNAi efficiency. The experimental system used can also influence the determination of the optimal dsRNA length. In the tobacco hornworm (*Manduca sexta*), oral delivery of a 2222 bp long dsRNA produced the strongest lethal effect, whereas the same fragment became ineffective when expressed in plant plastids because of reduced accumulation levels [18]. Conversely, shorter RNA constructs may, under certain conditions, achieve effects comparable to those of longer fragments. In *Phyllotreta striolata*, 212–214 bp linear dsRNAs and 21–24 bp short paperclip RNAs produced similar mortality rates, ranging from 68% to 84% [19]. In *Frankliniella occidentalis*, dsRNAs longer than 300 bp demonstrated high insecticidal activity, and field spray application was achieved when they were delivered using layered double hydroxide nanoparticles [20]. Collectively, these findings indicate that insect responses to dsRNA length are species-specific and context-dependent. Consequently, the optimal dsRNA length needs to be systematically screened, with bioinformatics tools such as dsRIP used to assist dsRNA design [21]. Furthermore, systematic screening of multi-gradient dsRNAs targeting a specific gene in the target pest is of substantial theoretical significance and practical value for optimizing RNAi strategies. However, the length-dependent effects of dsRNA in *M. separata* and the influence of target-region position on RNAi efficiency have not yet been systematically reported, and the corresponding design principles remain unclear.

In this study, the chitinase gene *MsChi2* was cloned from *M. separata*. Based on bioinformatics analyses, dsRNAs of different lengths (60, 200, 500, and 1000 bp) were designed to systematically investigate the effect of dsRNA length on RNAi efficiency. Subsequently, using the identified optimal length, five dsRNA fragments covering different regions of the coding sequence (CDS) were designed to evaluate the influence of target position on RNAi efficacy. The effects of these dsRNA constructs on larval body weight, mortality, target gene expression, chitin content, and molting were evaluated through feeding assays. We hypothesized that both dsRNA length and target position would significantly affect RNAi efficiency, and that siRNA prediction algorithms could inform the rational design of effective dsRNA constructs. This study aims to establish a design framework for optimizing dsRNA parameters targeting *MsChi2*, thereby providing theoretical and technical support for RNAi-based precision pest management of *M. separata*.

## 2. Materials and Methods

### 2.1. Insect Rearing

Eggs of *M. separata* were procured from Beijing Generalpest Biotech Research Co., Ltd. (Beijing, China). The insects were reared at 25 ± 2 °C, 75% relative humidity, and a 14:10 h light:dark photoperiod on fresh maize leaves. These rearing conditions do not induce diapause in *M. separata*, ensuring normal non-diapause development throughout the bioassays. Before bioassays, *M. separata* larvae were transferred to freshly prepared artificial diet, and third-instar larvae of uniform size (body weight 0.020 ± 0.002 g) and active feeding behavior were selected for all bioassays. Larvae displaying abnormal morphology or sluggish movement were excluded before treatment.

### 2.2. Domain Prediction and Phylogenetic Tree Construction

The conserved domains of the protein encoded by MsChi2 were predicted using the SMART online server (version 8.0, http://smart.embl-heidelberg.de/, accessed on 28 August 2026) with default parameters, and the composition and distribution of the signal peptide and functional domains were analyzed. Amino acid sequences of homologous chitinases from several lepidopteran insects were retrieved from the NCBI database. Multiple sequence alignment was performed using MUSCLE implemented in MEGA software (version 11.0.13), and a neighbor-joining phylogenetic tree was constructed using the Jones-Taylor-Thornton (JTT) model with 1000 bootstrap replicates to evaluate branch support. The evolutionary relationships of the target gene were analyzed in combination with taxonomic classification and the taxonomic status of the species.

### 2.3. Amplification of Target Gene Fragments and Construction of Recombinant Cloning Vectors

Total RNA was extracted from the gut of *M. separata* using TRIzol reagent (CWBio, Beijing, China). After genomic DNA removal, first-strand complementary DNA (cDNA) was synthesized with the HiScript II Q RT SuperMix for quantitative PCR (qPCR) (+gDNA wiper) kit (Vazyme, Nanjing, China). The cDNA served as a template for PCR amplification using *MsChi2*-specific primers listed in Table S1. The PCR program consisted of an initial denaturation at 95 °C for 3 min, followed by 35 cycles of 95 °C for 30 s, 58 °C for 30 s, and 72 °C for 1 min, with a final extension at 72 °C for 10 min. The amplified products were verified by agarose gel electrophoresis, after which four target fragments of different lengths were purified. Each purified fragment was ligated into the pMD19-T vector and transformed into DH5α competent cells. Recombinant plasmids were extracted and subjected to double digestion with *Hin*dIII and *Bam*HI (Thermo Fisher Scientific, Waltham, MA, USA), yielding four pMD19-T-*MsChi2* constructs of varying lengths. Positive clones were further identified by colony PCR using the same *MsChi2*-specific primers, followed by plasmid extraction and PCR confirmation. A second double-digestion with *Hin*dIII and *Bam*HI confirmed the presence and correct orientation of the inserts. Finally, all recombinant constructs were confirmed by Sanger sequencing (Sangon Biotech, Shanghai, China).

### 2.4. In Vitro Synthesis of dsRNA

To investigate the effect of dsRNA length on RNAi efficiency, four dsRNA fragments of 60, 200, 500, and 1000 bp were designed along the coding sequence (CDS) of MsChi2. The GC content of all fragments was controlled at approximately 50% to eliminate GC-bias as a confounding variable. The 60 bp fragment, representing the minimum length expected to generate functional siRNAs (theoretically yielding 2–3 siRNAs upon Dicer cleavage), served as a short-dsRNA control. The 200 bp fragment was designed to target an siRNA “hotspot” region previously identified in our small RNA sequencing study). The 500 bp fragment was designed to cover the N-terminal portion of the CDS, while the 1000 bp fragment was designed as an extended dsRNA to test whether longer fragments confer superior RNAi effects, spanning the central portion including the PKD domain region.

PCR amplification was performed using the sequence-verified recombinant plasmids as templates (Table S1). After the expected bands were confirmed by electrophoresis, PCR products were recovered and purified. In vitro transcription was conducted according to the instructions of the T_7_ RNA Transcription Kit Plus. The 20 μL reaction system consisted of T_7_ RNA polymerase, transcription buffer, NTP mix, and 1 μg of DNA template. The reaction was incubated at 37 °C for 2–4 h, followed by treatment at 72 °C for 10 min and gradual annealing at room temperature for 20 min to generate dsRNA. After transcription, the DNA template was removed by DNase I (1 U/μg DNA, 37 °C for 15 min), and single-stranded RNA was digested with RNase A (0.2 μg/mL, 37 °C for 20 min). The resulting dsRNA was purified by phenol-chloroform extraction followed by ethanol precipitation. The dsRNA yield was determined using a NanoDrop 8000 spectrophotometer (Thermo, USA), and its integrity was verified by RNA gel electrophoresis.

### 2.5. Feeding Assay

Third-instar larvae of *M. separata* with uniform body weight (0.020 ± 0.002 g) were selected for the feeding bioassays. After 12 h of starvation, the larvae were randomly divided into five groups: A blank control group (fed dsGFP) and four experimental groups that received dsRNAs of 60, 200, 500, and 1000 bp in length. Although the 200 bp group was subsequently subdivided into five treatment subgroups, it was considered a single treatment group in this experiment. Each treatment group contained 60 larvae in total, divided among three biological replicates (20 larvae per replicate). The control and experimental groups were fed artificial diets supplemented with dsRNAs of different lengths, with a daily dsRNA dose of 3 μg. Fresh dsRNA-containing diet was provided daily for seven consecutive days. Larval mortality was recorded every two days. At the end of the feeding trial, the body weight of surviving larvae was measured, and the expression level of the target gene, *MsChi2*, was quantified by quantitative reverse transcription PCR (qRT-PCR).

### 2.6. qRT-PCR

Total RNA was extracted from the gut of *M. separata* using TRIzol reagent from CWBIO (Beijing, China). Following genomic DNA removal, first-strand cDNA was synthesized using the HiScript II Q RT SuperMix for qPCR (+gDNA wiper) kit (Vazyme, Nanjing, China). The *MsActin* gene (accession No. GQ856238.1) from *M. separata* was selected as the reference gene (Table S1), and qRT-PCR was performed using ChamQ SYBR qPCR Master Mix from Vazyme. Each 10 μL reaction contained 5 μL of 2× premix, 0.25 μL each of forward and reverse primers (10 μM), 1 μL of cDNA template, and nuclease-free water to a final volume of 10 μL. The amplification protocol consisted of an initial denaturation at 95 °C for 2 min, followed by 40 cycles of 95 °C for 5 s, 60 °C for 30 s, and 72 °C for 30 s, after which melting-curve analysis was performed. The relative expression level of the target gene was calculated using the 2^−ΔΔCT^ method. All experiments were performed with three biological replicates.

### 2.7. Chitin Content Assay

The chitin content of the insect body was determined using a Sigma-Aldrich chitin assay kit (MAK242). Fresh larvae were ground in liquid nitrogen, and 50–100 mg of powder was accurately weighed. Lipids were removed by washing with chloroform/methanol (1:1, *v*/*v*), and proteins were digested with 1 M NaOH at 80 °C for 30 min. The resulting chitin pellet was hydrolyzed in 6 M HCl at 100 °C for 6 h to release N-acetyl-D-glucosamine (GlcNAc). After neutralization, the colorimetric working solution was added, and the samples were incubated in the dark. Absorbance was measured at 530 nm, and quantification was performed using a GlcNAc standard curve. Additionally, chitin content in each sample was calculated according to the following formula: Chitin content (μg/g) = (*C* × *V* × *D*/*W*) × 0.9. In this formula, *C* represents the GlcNAc concentration, *V* the total reaction volume, *D* the dilution factor, *W* the fresh weight of the sample, and 0.9 the conversion coefficient. Three biological replicates were included for each group.

### 2.8. Statistical Analysis

Candidate effective siRNA sequences were predicted using DNAMAN (version 9.0, Lynnon Biosoft, Vaudreuil-Dorion, Quebec, Canada). All continuous variables (larval body weight, relative gene expression, and chitin content) were first tested for normality of distribution using the Shapiro–Wilk test, and homogeneity of variances among groups was assessed using Levene’s test. After confirming normality and homogeneity of variances, these data were analysed by one-way analysis of variance (ANOVA) followed by Bonferroni’s post hoc test for multiple pairwise comparisons, or by Dunnett’s test when only comparisons against the control group were of interest, using IBM SPSS Statistics software (version 19.0, SPSS Inc., Chicago, IL, USA). For comparisons between only two groups, independent-samples *t*-tests were performed after validating normality and variance homogeneity. For survival data, Kaplan–Meier survival curves were constructed, and differences between treatment groups were assessed using the log-rank test. For proportional data such as mortality rates and percentages of abnormal individuals at the endpoint, chi-square tests were used to compare groups. Graphs were generated using GraphPad Prism software (version 7.0, GraphPad Software, San Diego, CA, USA). Data are presented as the mean ± SD as indicated in the figure legends. To account for baseline mortality observed in the control group, mortality rates of treatment groups were corrected using Abbott’s formula: corrected mortality (%) = (treatment mortality-control mortality)/(1-control mortality) × 100. Corrected mortality values were used in the evaluation of insecticidal efficacy.

## 3. Results

### 3.1. Bioinformatic Characteristics of the MsChi2 Gene

**Figure 1:**
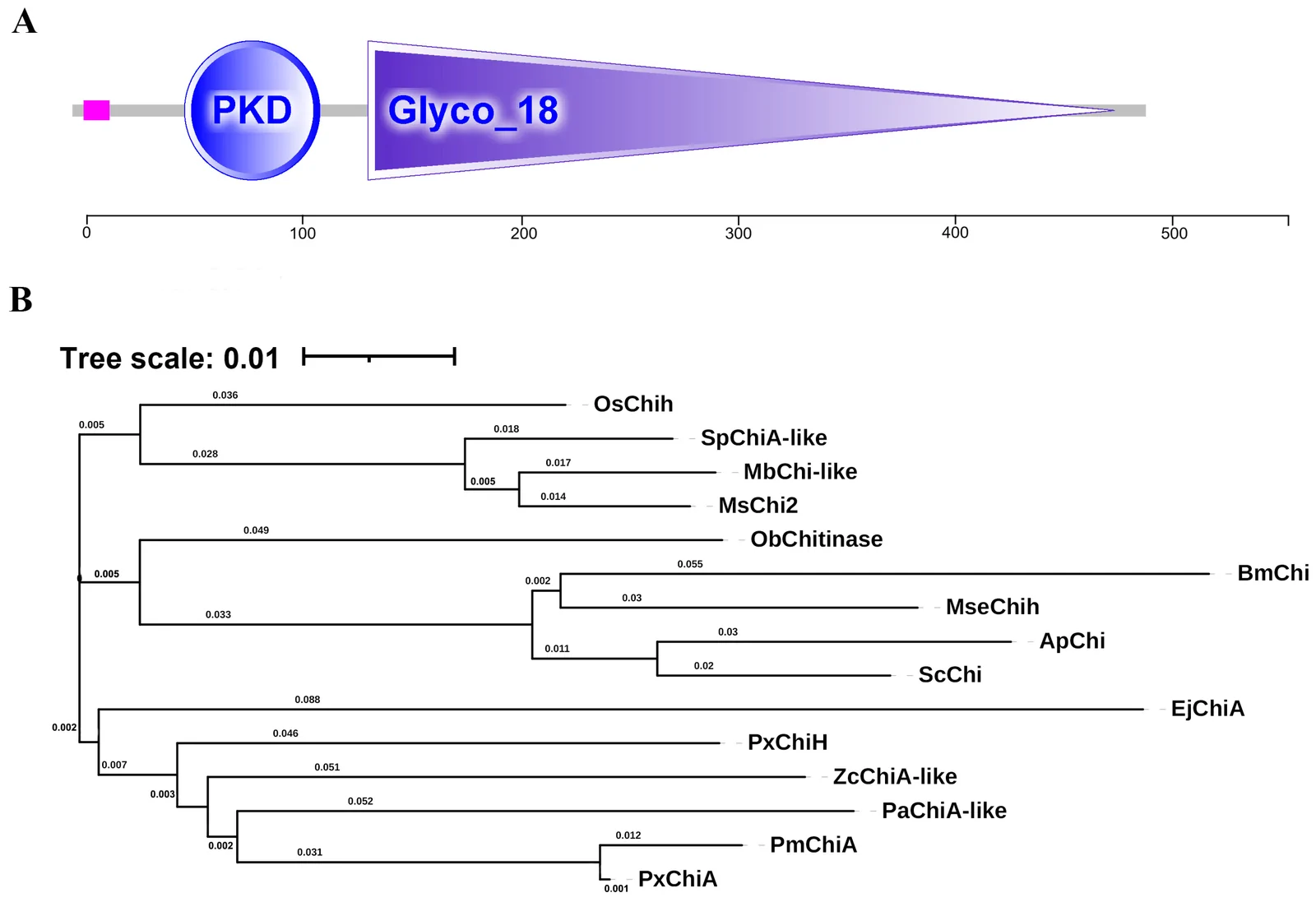
Domain features and phylogenetic analysis of the MsChi2 protein. (**A**) The conserved domains of MsChi2 consist of an N-terminal signal peptide, a central PKD domain, and a C-terminal Glyco_18 catalytic domain. (**B**) Neighbor-joining phylogenetic tree constructed based on the amino acid sequences of *M. separata* MsChi2 and chitinases from 13 lepidopteran insects. Numbers at the branch nodes indicate bootstrap values from 1000 replicates; the scale bar represents genetic distance (0.01). Species abbreviations: OsChiH (*Ostrinia scapulalis*), SpChiA-like (*Spodoptera frugiperda*), MbChi-like (*Mamestra brassicae*), ObChitinase (*Operophtera brumata*), BmChi (*Bombyx mori*), MseChiH (*Manduca sexta*), ApChi (*Antheraea pernyi*), ScChi (*Samia cynthia*), PxChiH (*Plutella xylostella*), ZcChiA-like (*Zerene cesonia*), PaChiA-like (*Papilio polyxenes*), PmChiA (*Papilio machaon*), PxChiA (*Papilio xuthus*), and EjChiA (*Eumeta japonica*).

Domain analysis indicated that the *MsChi2*-encoded protein comprises 555 amino acids and contains the typical structural features of GH18 family chitinases: An N-terminal signal peptide, a central polycystic kidney disease (PKD) carbohydrate-binding domain, and a highly conserved Glyco_18 catalytic domain at the C-terminus. These features indicate that the protein possesses complete substrate-binding and hydrolytic functions (Figure 1A). A phylogenetic tree constructed from chitinase amino acid sequences of 14 lepidopteran insects revealed that all sequences clustered into three major evolutionary branches, broadly consistent with their taxonomic relationships. The *M. separata* MsChi2 protein was most closely related to homologs in *Mamestra brassicae*, *Spodoptera frugiperda*, and *Ostrinia scapulalis*, with these sequences clustering within the same branch. The chitinases of the *Operophtera brumata*, *Bombyx mori*, *Manduca sexta*, *Antheraea pernyi*, and *Samia cynthia* formed a second branch. The chitinases of the *Plutella xylostella*, *Zerene cesonia*, *Papilio machaon*, *Papilio xuthus*, and *Eumeta japonica* formed the third branch (Figure 1B), indicating that chitinase genes are highly evolutionarily conserved among noctuid insects.

### 3.2. In Vitro Synthesis of dsRNAs of Different Lengths Targeting MsChi2

**Figure 2:**
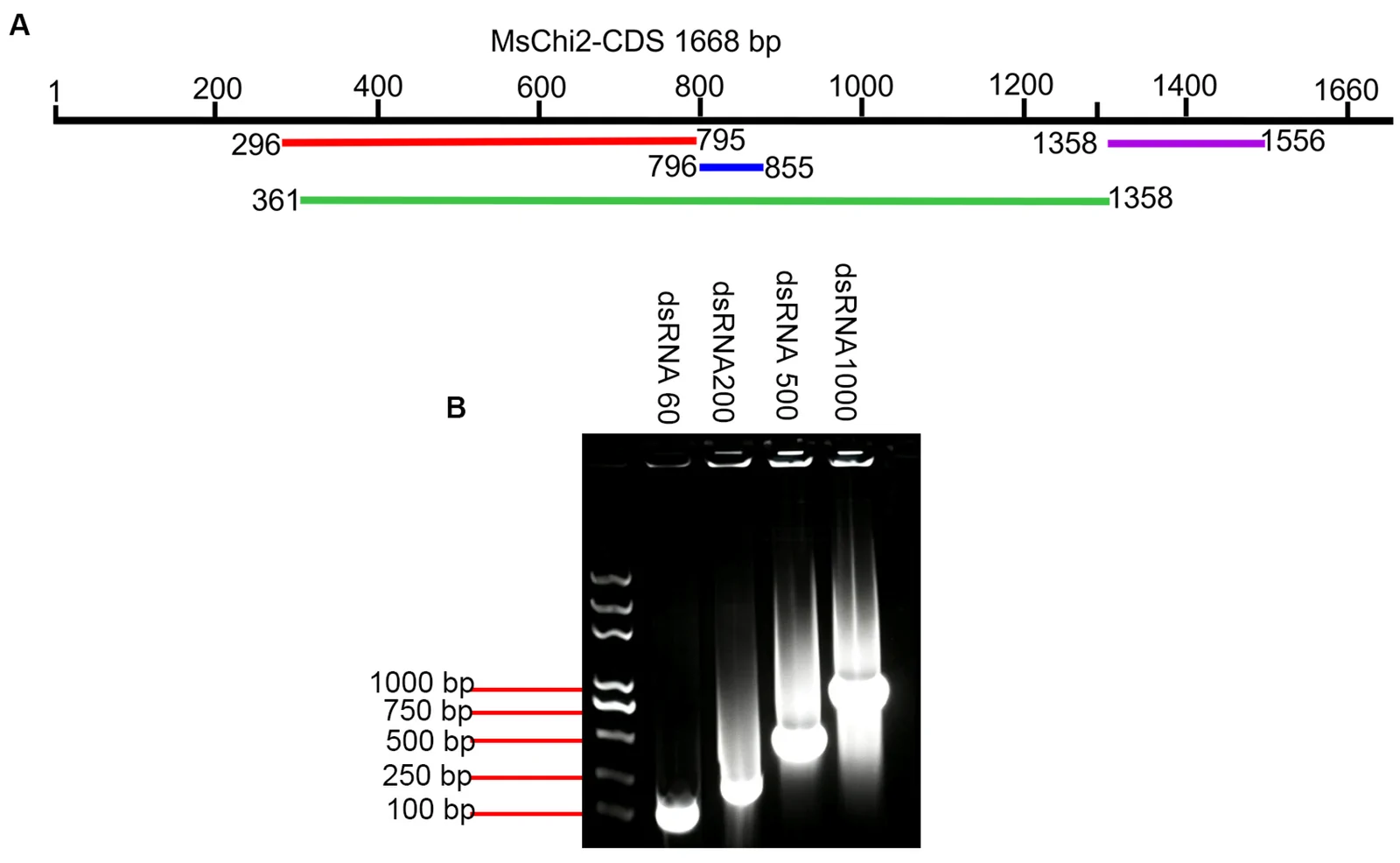
Schematic diagram of the design positions of *MsChi2*-specific dsRNAs and validation by agarose gel electrophoresis. (**A**) Schematic representation of the relative positions of dsRNAs of different lengths within the *MsChi2* CDS. Different colors indicate different fragment regions: dsRNA 60 covers 796–855 bp, dsRNA 200 covers 1358–1556 bp, dsRNA 500 covers 296–795 bp, and dsRNA 1000 covers 361–1358 bp. The GC content of each fragment was controlled at approximately 50%. (**B**) Agarose gel electrophoresis analysis of dsRNA products. M: Trans2K Plus Marker.

To systematically investigate the effect of dsRNA length on RNAi efficiency, four dsRNA fragments of 60, 200, 500, and 1000 bp were designed along the CDS of MsChi2, based on its conserved domains and following the design principles detailed in Section 2.4. These fragments were amplified, cloned into the pMD19-T vector, and verified by colony PCR, plasmid PCR, double digestion, and sequencing (Figure S1). Using the recombinant plasmids as templates, the four dsRNAs were synthesized in vitro with the MEGAscript T7 kit. As shown in Figure 2A, the fragments are distributed across the 1668 bp CDS, with GC content adjusted to approximately 50% to exclude any potential influence on dsRNA structural stability or RNAi efficiency. Agarose gel electrophoresis (Figure 2B) confirmed the purity and correct sizes of all products, each displaying a single clear band at the expected position (60, 200, 500, or 1000 bp), indicating successful synthesis without detectable degradation or nonspecific by-products.

### 3.3. Effects of dsRNAs of Different Lengths on the Growth and Development of M. separata Larvae

**Figure 3:**
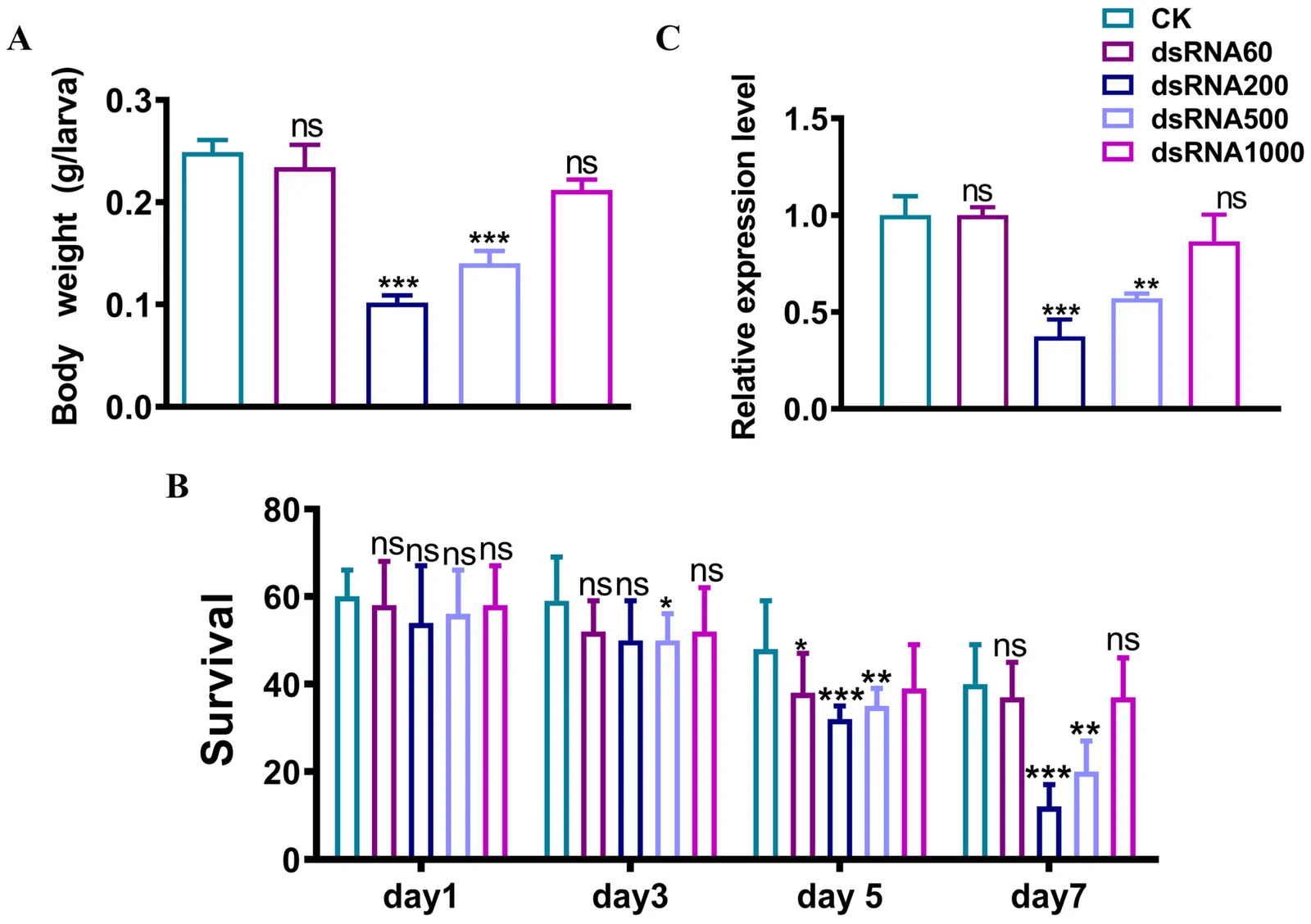
RNAi effects of feeding *M. separata* larvae with *MsChi2* dsRNAs of different lengths. (**A**) Larval body weight after seven days of feeding treatment (mean ± SD). (**B**) Cumulative survival rates at days 1, 3, 5, and 7 of the feeding period. Data are shown as means ± SD (*n* = 20 larvae per replicate, three biological replicates per treatment). Survival rates were derived from Kaplan–Meier estimates; mortality rates discussed in the text were calculated as 1-survival rate. (**C**) Relative expression level of the *MsChi2* gene, with the dsGFP control group used as the reference and set to 1; mean ± SD. Asterisks indicate significant differences compared with the dsGFP control (ns, not significant; *, *p* < 0.05; **, *p* < 0.01; ***, *p* < 0.001).

A total of 300 uniformly developed third-instar larvae were randomly divided into five groups. The larvae were fed artificial diets containing equal amounts (3 μg) of 60, 200, 500, or 1000 bp *MsChi2* dsRNA, respectively, while the control group was fed an equal amount of dsGFP. After seven days of continuous treatment, larval body weight in the 200 and 500 bp dsRNA treatment groups was significantly lower than that in the control group, whereas no significant difference was observed between 60 or 1000 bp dsRNA treatment groups and the control group (Figure 3A). Larval mortality gradually increased over time. On day 7, mortality reached 65.9% in the 200 bp treatment group and 51.2% in the 500 bp treatment group (calculated as 1-survival rate from the Kaplan–Meier estimates). Both values were significantly higher than the 18% mortality observed in the control group, while 60 and 1000 bp treatment groups demonstrated no significant difference from the control group (Figure 3B). The qRT-PCR analysis demonstrated that *MsChi2* expression was strongly downregulated in the 200 bp dsRNA treatment group, followed by the 500 bp treatment group. Conversely, 60- and 1000 bp dsRNAs produced no significant reduction in target gene expression (Figure 3C). Collectively, the 200 bp dsRNA produced the strongest effects in inhibiting larval growth, inducing mortality, and silencing the target gene.

### 3.4. Effects of dsRNAs of Different Lengths on Larval Chitin Content and Molting Phenotype

**Figure 4:**
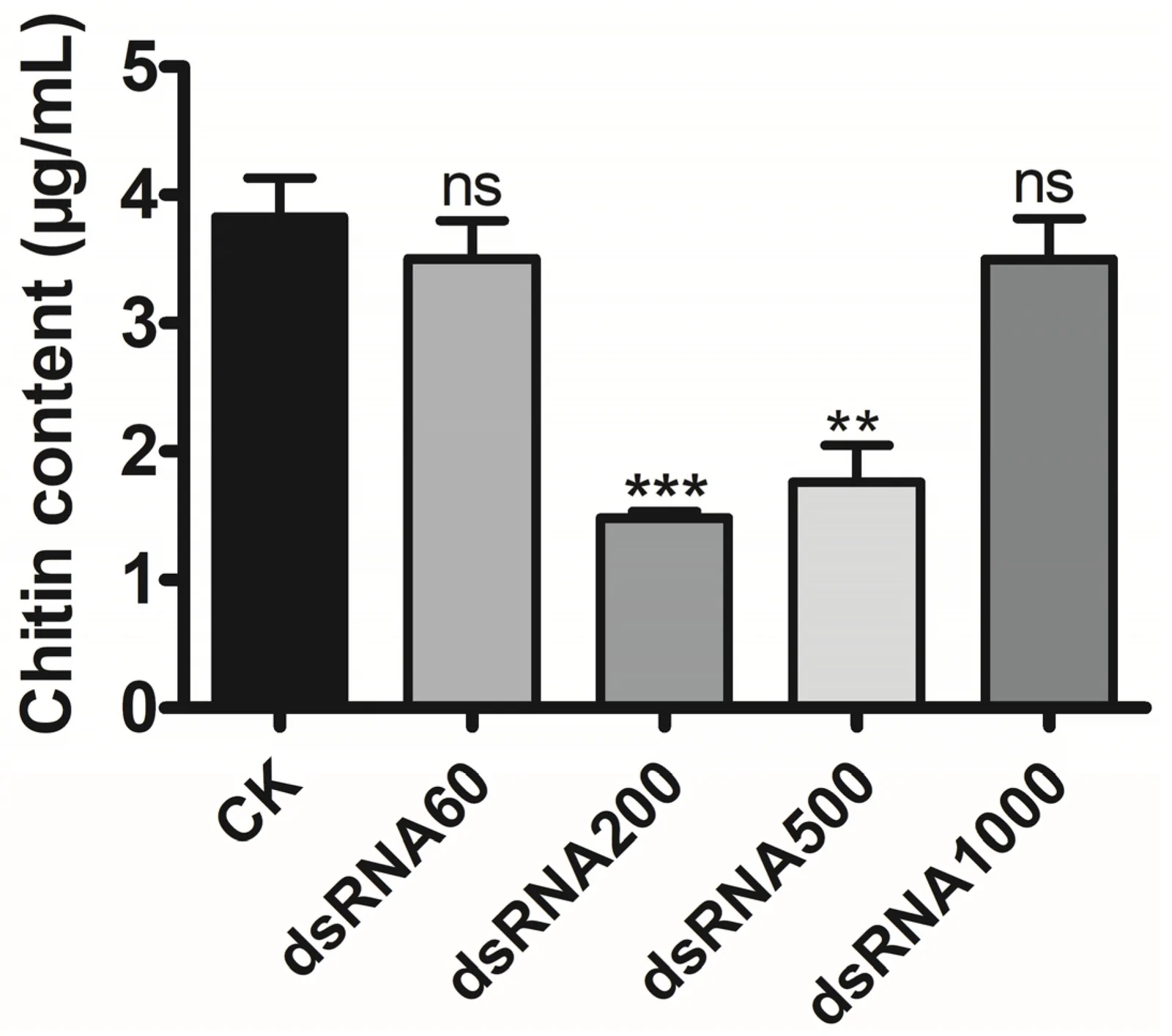
Changes in chitin content in *M. separata* larvae after feeding with *MsChi2* dsRNAs of different lengths. The x-axis indicates the experimental groups: CK represents the blank control group, whereas dsRNA60, dsRNA200, dsRNA500, and dsRNA1000 represent treatment groups fed 60, 200, 500, and 1000 bp *MsChi2* dsRNAs, respectively. The y-axis indicates larval chitin content (μg/mL). All data are presented as mean ± SD. Differences between each treatment group and the control group were analyzed using one-way ANOVA with Dunnett’s post hoc test (ns, not significant; **, *p* < 0.01; ***, *p* < 0.001).

Feeding treatment with *MsChi2* dsRNAs of different lengths significantly altered the level of chitin accumulation in *M. separata* larvae. The chitin content measured in each group is illustrated in Figure 4. Compared with the control group, chitin content indicated no significant change in either the dsRNA60 or dsRNA1000 treatment group, indicating that these fragments failed to effectively interfere with chitin metabolism. Conversely, chitin content was reduced by 61.5% and 54% in the dsRNA200 and dsRNA500 treatment groups, respectively, relative to the control group. Both reductions were statistically significant, with the greatest reduction observed in the dsRNA200 treatment group. These results are consistent with the gene expression levels (Figure 3C), supporting the conclusion that 200 and 500 bp *MsChi2* dsRNAs effectively suppressed target gene expression and consequently inhibited chitin synthesis or accumulation in *M. separata*. Conversely, 60 and 1000 bp dsRNAs exhibited non-significant biological effects in this experimental system.

**Figure 5:**
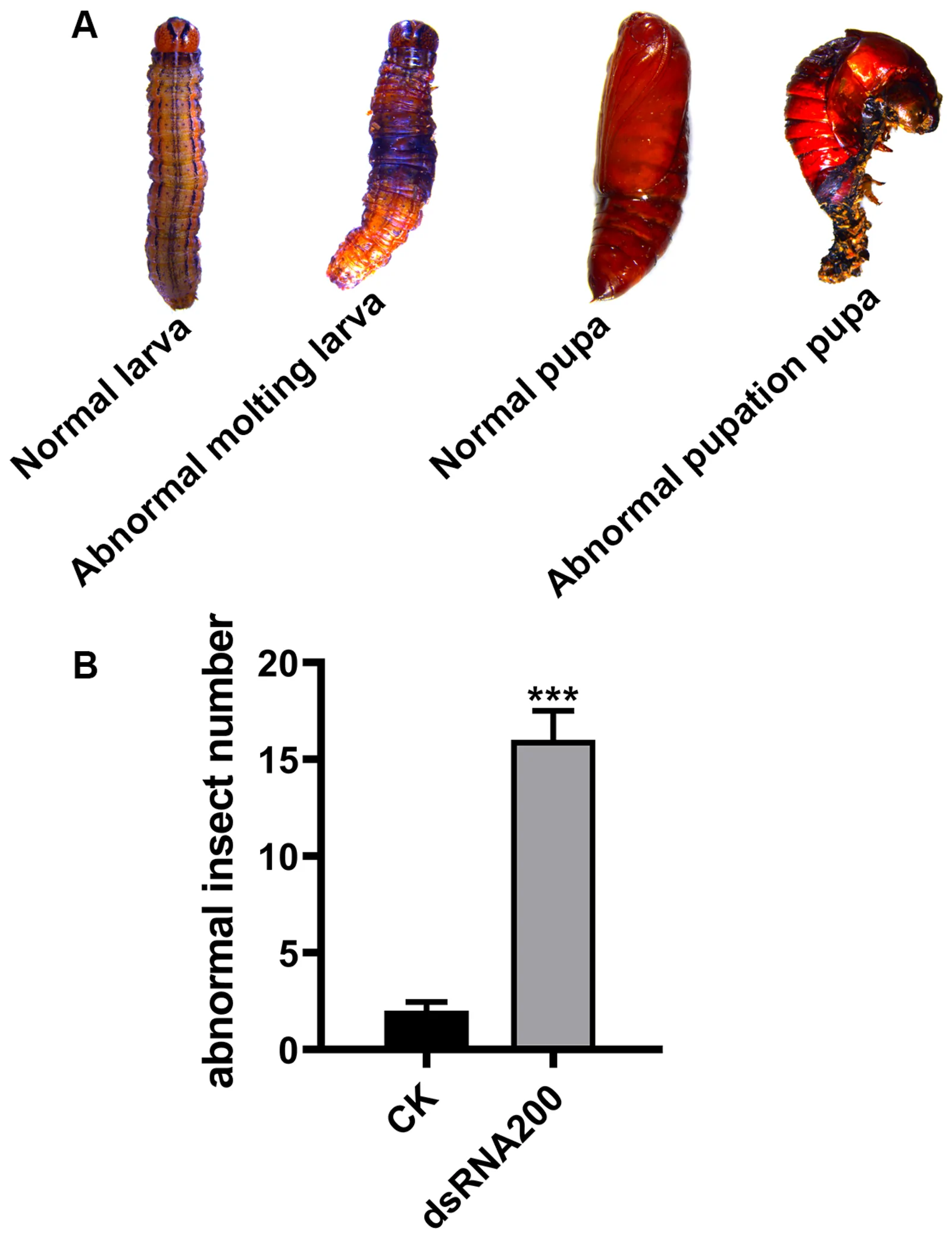
Effects of 200 bp dsRNA treatment on larval molting and pupation in *M. separata*. (**A**) Representative photographs of larval and pupal morphology in different treatment groups. (**B**) Statistical comparison of the numbers of abnormal insects in different treatment groups. The dsGFP was the negative control group, while dsRNA200 was the 200 bp dsRNA treatment group. Data are presented as mean ± SD (*n* = 3), ***, *p* < 0.001 (*t*-test). The scale represents the actual number of abnormal individuals in each group.

To complement the chitin content analysis, morphological observations and quantification of developmental abnormalities were performed for the dsRNA200-treated group and the dsGFP control group of *M. separata*. The results are illustrated in Figure 5. Larvae in the control group exhibited smooth body surfaces, clearly defined body segments, and completed pupation to produce spindle-shaped pupae with uniform coloration. Conversely, larvae treated with the 200 bp dsRNA displayed a range of developmental abnormalities, including incomplete shedding of the old cuticle, distorted body segments, localized wrinkled cuticle, cuticle darkening, malformed pupae, and failure to complete normal pupation (Figure 5A). The numbers of developmentally abnormal individuals in each treatment group were quantified. The results (Figure 5B) indicated that, compared with the dsGFP control group, the number of abnormal insects in the dsRNA200-treated group increased significantly. These observations further indicated that treatment with 200 bp *MsChi2* dsRNA effectively disrupts the normal physiological processes associated with chitin metabolism in *M. separata*. This process leads to severe developmental defects during larval molting and pupation, confirming the critical role of the *MsChi2* gene in insect metamorphosis.

### 3.5. Diversified dsRNA Design Targeting the MsChi2 Gene of M. separata and Analysis of Corresponding Candidate siRNAs

**Figure 6:**
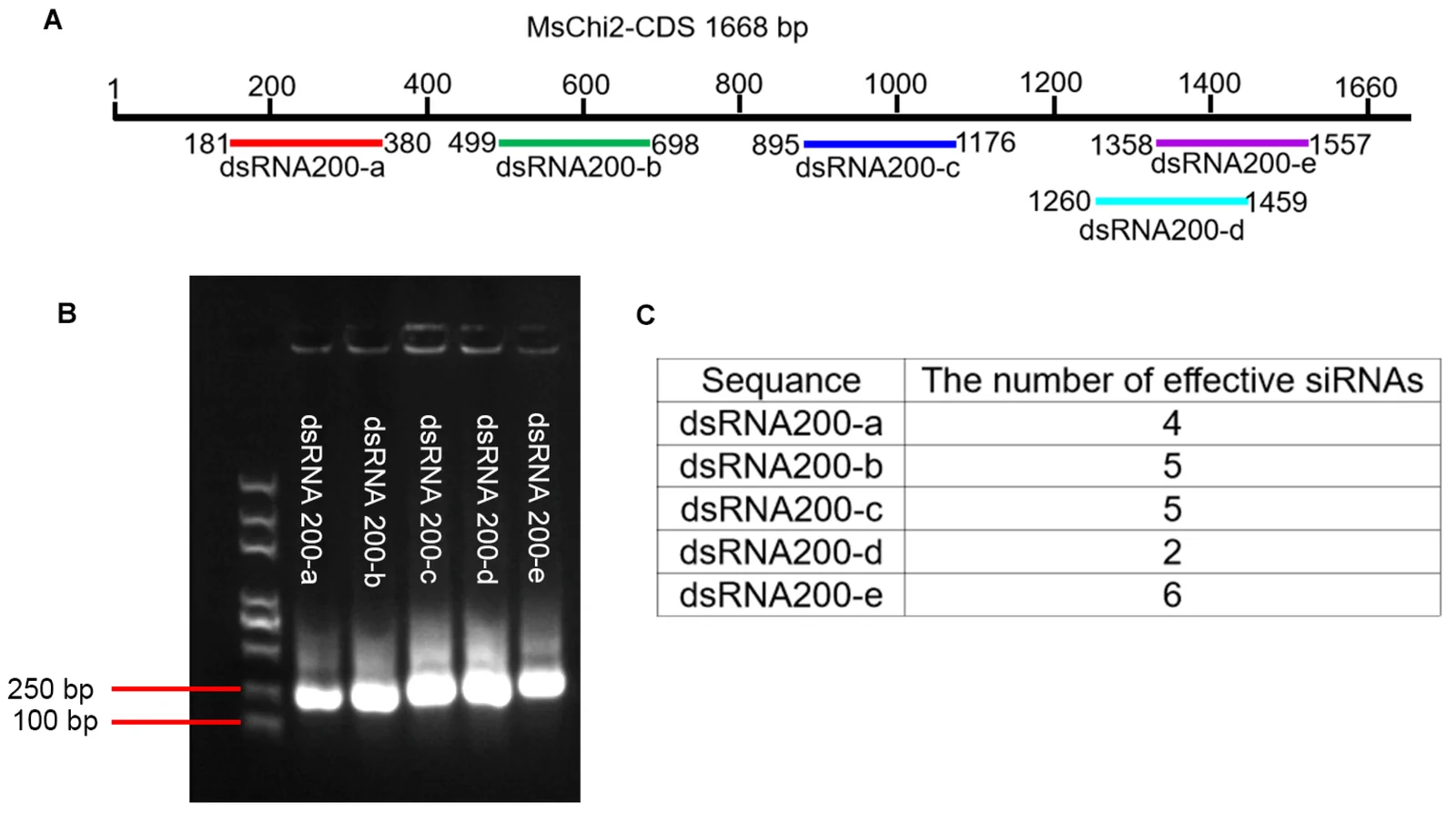
Schematic diagram of different dsRNA200 fragments designed within the CDS region of *MsChi2* gene, electrophoretic validation, and statistics of the numbers of effective siRNAs. (**A**) Schematic representation of the full-length CDS of *MsChi2* gene (1668 bp), indicating the positions covered by each dsRNA fragment: dsRNA200-a (181–380 bp), dsRNA200-b (499–698 bp), dsRNA200-c (895–1176 bp), dsRNA200-d (1260–1459 bp), and dsRNA200-e (1358–1557 bp). (**B**) Agarose gel electrophoresis of the five dsRNA fragments after in vitro transcription and synthesis. M: Trans2K Plus Marker. (**C**) Summary of the numbers of candidate effective siRNAs generated by each dsRNA.

In the preceding experiments, dsRNAs of different lengths targeting the *MsChi2* gene, including 60, 200, 500, and 1000 bp fragments, were compared, with the 200 bp dsRNA producing the highest RNAi efficiency. To further investigate the effect of dsRNA target position on RNAi efficiency, four additional 200 bp dsRNA fragments were designed within the CDS of this gene, which has a full length of 1668 bp. These fragments were designated dsRNA200-a, covering 181–380 bp; dsRNA200-b, covering 499–698 bp; dsRNA200-c, covering 895–1176 bp; dsRNA200-d, covering 1260–1459 bp. The previously validated dsRNA200 fragment, which produced effective RNAi, covered 1358–1557 bp and was redesignated as dsRNA200-e (Figure 6A). To minimize potential variation arising from GC content and other sequence-dependent properties, all fragments were selected to have GC contents ranging from 50% to 53%. After in vitro transcription and synthesis, agarose gel electrophoresis demonstrated that all dsRNA fragments produced single bands of the expected size (Figure 6B).

All dsRNA200 fragments were analyzed using DNAMAN software to predict the number of candidate effective siRNAs generated from each construct. The number of effective siRNAs differed significantly among dsRNAs targeting different positions. The dsRNA200-a was predicted to generate four effective siRNAs; dsRNA200-b and dsRNA200-c each generated five effective siRNAs; dsRNA200-d generated only two effective siRNAs; whereas dsRNA200-e generated six effective siRNAs, the highest number among all tested fragments (Figure 6C). The relative locations of the predicted effective siRNAs within each fragment are illustrated in Figure S2. These results indicate that dsRNA200-b, dsRNA200-c, and dsRNA200-e have relatively high potential RNAi efficacy.

### 3.6. Effects of dsRNA200 Fragments Targeting Different Positions on RNAi Efficiency in M. separata Larvae

**Figure 7:**
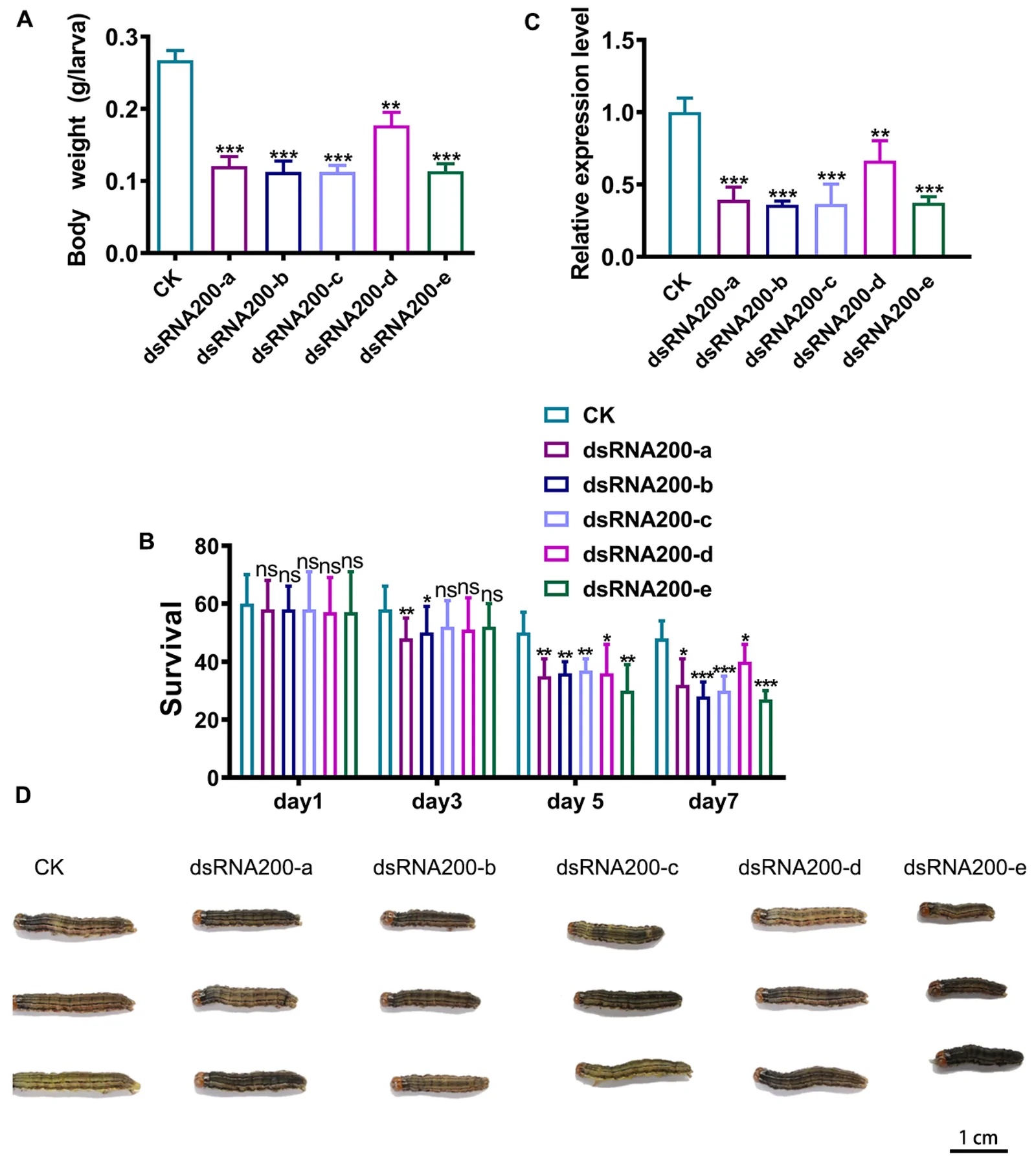
RNAi effects in *M. separata* larvae fed in vitro-synthesized 200 bp dsRNAs targeting different positions. (**A**) Body weight of *M. separata* larvae in the control group (CK) and in groups treated with 200 bp dsRNAs targeting different positions after seven days of feeding (unit: g). Data are presented as mean ± SD. Asterisks indicate significant differences compared with the dsGFP control (ns, not significant; *, *p* < 0.05; **, *p* < 0.01; ***, *p* < 0.001). (**B**) Changes in survival rates of *M. separata* larvae in different treatment groups on days 1, 3, 5, and 7 of feeding. Data are presented as mean ± SD. (**C**) Relative expression levels of the target gene *MsChi2* in each treatment group as determined by qRT-PCR. Gene expression in larvae of the blank control group fed dsGFP was used as the reference and set to 1. Data are presented as mean ± SD (*n* = 3). (**D**) Representative larval phenotypes in the groups treated with 200 bp dsRNAs targeting different positions after seven days of feeding. Scale bar = 1 cm.

To investigate whether dsRNAs targeting distinct regions of the same gene affect RNAi efficiency, five 200 bp dsRNAs covering different coding segments of *MsChi2* were synthesized and delivered to third-instar *Mythimna separata* larvae via artificial diet feeding. Larvae fed with dsGFP served as the control group, while dsRNA200-e was the fragment confirmed to trigger effective RNAi in our previous laboratory work. After continuous feeding for seven days, larval body weights in all dsRNA treatment groups were significantly lower than those in the control group (Figure 7A). Notably, dsRNA200-a, -b, -c and -e induced comparable and strong growth inhibition. Although the suppressive effect of dsRNA200-d was still statistically significant relative to the control, its efficacy was consistently weaker than that of the other four fragments. Survival monitoring throughout the feeding period revealed time-dependent increases in larval mortality for all treatment groups. Significant differences in survival rates between all dsRNA treatments and the control were observed starting from day 3. By day 7, survival rates of all treatment groups were significantly lower than the control (Figure 7B), indicating that impaired larval growth ultimately translated into elevated mortality. The qRT-PCR analysis conducted on day seven validated the phenotypic observations. The transcription level of *MsChi2* was significantly downregulated in all larvae administered dsRNA compared with the control. Among the five dsRNA fragments, dsRNA200-a, -b, -c, and -e produced the strongest target gene silencing. While dsRNA200-d significantly suppressed target gene expression, the inhibitory magnitude was relatively moderate (Figure 7C). These molecular data were fully consistent with morphological observations: Larvae treated with dsRNA200-a, -b, -c and -e exhibited severe growth retardation and clear phenotypic abnormalities, whereas mild developmental defects were detected in larvae from the dsRNA200-d group (Figure 7D).

Collectively, these results demonstrate that all five tested 200 bp dsRNAs targeting different regions of *MsChi2* can trigger RNAi responses in *M. separata* larvae. However, RNAi efficiency differed markedly among target sites. Notably, dsRNA200-a, -b, -c and -e act as highly efficient RNAi triggers, while dsRNA200-d displays relatively limited potency for functional research of this gene.

## 4. Discussion

*M. separata* causes substantial economic losses to staple crops, indicating the need for developing environmentally friendly pest control technologies [22]. In this study, the GH18 family chitinase gene *MsChi2* was cloned and identified from *M. separata*. The effects of dsRNA fragment length and target position on the silencing efficiency of this gene were systematically investigated, together with their impacts on larval chitin content and molting phenotypes. These results provide a foundation for optimizing dsRNA design parameters in RNAi-based management of this pest.

Bioinformatics analysis indicated that the *MsChi2*-encoded protein possesses the characteristic domain architecture of GH18 chitinases, including an N-terminal signal peptide, a central PKD domain, and a C-terminal Glyco_18 catalytic domain [23]. Phylogenetic analysis revealed that MsChi2 shares the greatest sequence similarity with homologous proteins from noctuid species such as *Mamestra brassicae* and *Spodoptera frugiperda*. However, it is more divergent from those of *Bombyx mori* and *Manduca sexta*, reflecting the evolutionary conservation of this gene within Noctuidae. The high conservation of chitinase genes makes them potentially ideal targets for RNAi-based pest control. Silencing chitin synthase genes in *Chilo suppressalis* and *Cnaphalocrocis medinalis* can significantly inhibit larval molting and pupation [24,25]. Additionally, bacterial expression systems, recombinant plant viruses, and in vitro-synthesized dsRNA have all been successfully used to suppress *MsChi1* and *MsChi2* in *M. separata*, resulting in reduced larval body weight and increased mortality. Together, these studies establish the feasibility of RNAi as a strategy for controlling *M. separata* [12,13,14].

Despite these advances, previous studies generally evaluated only a single dsRNA or a single target site, and thus did not systematically evaluate the influence of molecular design parameters. Using small RNA sequencing, Bao et al. (2021) identified distinct siRNA “hotspot regions” within *MsChi2*, suggesting that target-site selection may be a key factor affecting RNAi efficiency [26]. On this basis, the present study used *MsChi2* as the target gene to systematically compare, for the first time, the RNAi effects of dsRNAs with different lengths (60, 200, 500, and 1000 bp) and different target positions (five 200 bp fragments), to determine the optimal design parameters.

Comparison of dsRNAs of different lengths demonstrated that the 200 and 500 bp dsRNAs produced the strongest inhibitory effects on larval growth and survival. The dsRNA200 treatment group achieved a 59% reduction in larval body weight, 65.9% mortality, and 62% suppression of *MsChi2* expression, whereas 60- and 1000 bp dsRNAs exhibited only limited RNAi activity. In our experimental system, the 18% mortality observed in the control group over the 7-day feeding period is within the acceptable range for long-term oral bioassays using artificial diets, where baseline mortality of 10–20% is commonly attributed to natural individual variation and mechanical injury during daily diet replacement. To exclude the influence of this non-specific background mortality, Abbott’s correction was applied. After correction, the 200 bp dsRNA still induced 65.9% net mortality and the 500 bp dsRNA induced 51.2% net mortality, confirming that the observed effects were attributable to specific silencing of *MsChi2* rather than non-specific factors. These results demonstrate that dsRNA length is a critical factor affecting RNAi efficiency and that an optimal length window exists, rather than a simple monotonic increase in efficiency with increasing fragment length. This finding is consistent with previous reports indicating that dsRNA length effects are species- and gene-specific, with optimal lengths often falling within a defined range rather than increasing indefinitely [15,17,20]. Consequently, the optimal dsRNA length should be determined experimentally for each specific species and target gene.

The limited RNAi activity observed with the 60 bp dsRNA may be explained by several factors. Short dsRNAs generate only 2–3 siRNAs upon Dicer cleavage and are highly susceptible to degradation by midgut nucleases [27,28]. Conversely, the poor performance of the 1000 bp dsRNA is inconsistent with the intuitive assumption that “longer is better”. Several mechanisms may contribute to this phenomenon. First, Longer RNA molecules are more likely to form complex secondary structures that hinder complete annealing, thereby reducing the effective dsRNA concentration [15]. Second, the intracellular transport of dsRNA in lepidopteran cells is inefficient, and larger molecules may further impede cytoplasmic delivery [29]. Third, excessively long dsRNAs may hinder efficient unwinding and processing by Dicer because of their structural diversity [26]. Additionally, longer dsRNAs may be more susceptible to incomplete processing by nucleases in the hemolymph or gut lumen, generating non-functional degradation products. Collectively, these findings indicate that a fragment length of 200–500 bp represents an effective length range, with the 200 bp construct exhibiting the strongest overall performance under the experimental conditions.

After identifying 200 bp as the optimal RNAi-inducing length, this study further designed five 200 bp dsRNA fragments targeting different positions within the CDS region of the *MsChi2* gene to investigate the influence of the target site on RNAi efficiency. The fragments differed markedly in their effects on larval body weight, survival, and target gene expression. Specifically, dsRNA200-a, dsRNA200-b, dsRNA200-c, and dsRNA200-e exhibited strong inhibitory activity, whereas dsRNA200-d demonstrated a relatively weak effect. This positional effect is consistent with previous studies demonstrating that Dicer-processable sites are unevenly distributed along target sequences and that only a subset of cleavage sites generates highly effective siRNAs [26,30,31]. The siRNA prediction analysis indicated that dsRNA200-d generated only two effective siRNAs, far fewer than the other fragments, which produced 4–6 effective siRNAs. This prediction closely paralleled the observed differences in biological activity. Additionally, sequence features, such as GC content and local secondary structure, may influence Dicer processing efficiency. Furthermore, fragments predicted to generate relatively few effective siRNAs may still retain partial silencing activity, suggesting the presence of functional siRNAs that were not predicted [15]. Consequently, dsRNA design should optimize fragment length and incorporate siRNA prediction algorithms to preferentially select target sites capable of generating a larger number of high-quality siRNAs, improving RNAi efficacy.

In this study, changes in larval chitin content were further measured after feeding with *MsChi2* dsRNAs of different lengths. The chitin content assay revealed that chitin content was significantly reduced in the 200 and 500 bp treatment groups, whereas no significant difference was observed in the 60 and 1000 bp groups. Morphological observations confirmed that larvae in the dsRNA200 treatment group exhibited severe molting defects, including incomplete shedding of the old cuticle, distorted body segments, and abnormal pupation characterized by malformed pupae and incomplete sclerotization. The number of abnormal individuals was significantly higher than that in the control group, providing direct evidence that *MsChi2* plays a key regulatory role in chitin metabolism in *M. separata*. This is consistent with RNAi studies of chitinase genes in *Chilo suppressalis* and *Tribolium castaneum* [25,32], confirming that *MsChi2* is a critical gene for growth and development and has a biological basis as a target for RNAi-based control.

Despite the promising results, several limitations of this study should be addressed. First, dsRNA was delivered by feeding larvae an artificial diet containing dsRNA. However, highly active dsRNA-degrading enzymes, or dsRNases, are present in the midgut of Lepidoptera, and these enzymes are considered one of the key factors underlying the generally low RNAi efficiency observed in lepidopteran insects [33,34]. In future studies, liposome encapsulation or nanomaterial-mediated delivery could be used to protect dsRNA from degradation [35,36]. Second, this study evaluated RNAi effects only during the larval stage and did not examine long-term effects such as adult fecundity and offspring development. In the future, *MsChi2* dsRNA could be integrated into transgenic maize plants to achieve sustained pest control and promote the transition of RNAi technology from laboratory research to field application.

In summary, this study characterized the GH18 chitinase gene *MsChi2* from *M. separata* and systematically evaluated the effects of dsRNA length and target position on RNAi efficiency. The results identified 200 bp as the optimal dsRNA length and demonstrated a positive association between the predicted number of effective siRNAs and RNAi performance. Furthermore, *MsChi2* was confirmed to play an essential role in chitin metabolism, larval development, and molting. These findings enrich the theoretical understanding of RNAi targeting chitinase genes in Lepidoptera and provide important experimental evidence and technical parameters for RNAi-based precision management of *M. separata*.

## 5. Conclusions

In conclusion, this study characterized the GH18 chitinase gene *MsChi2* from *M. separata* and demonstrated that both dsRNA length and target position critically influence RNAi efficacy against this gene. Among the tested constructs, the 200 bp dsRNA length exhibited the highest RNAi efficiency, and the correlation between predicted siRNA numbers and phenotypic effects provides a rational design framework for dsRNA development. These findings identify *MsChi2* as a promising RNAi target for pest control. Future research should focus on improving dsRNA delivery systems, including nanoparticle-based formulations and transgenic plant expression systems, to enable sustainable field application against *M. separata*.

## Data Availability

All raw qPCR data, agarose gel images, primer sequences and siRNA prediction data generated in this study are included within the article and Supplementary Materials. Further original experimental datasets are available from the corresponding author upon reasonable request.

## Supplementary Materials

The following supporting information can be downloaded at: https://www.mdpi.com/article/10.3390/insects17090931/s1, Table S1: Primer names and sequences used in this study. Figure S1: Amplification of *MsChi2* fragments of different lengths and identification of recombinant plasmids. Figure S2: Prediction of the relative locations and numbers of effective siRNA candidates within the five 200 bp dsRNA fragments using DNAMAN software.
