# Supplementary material for: Optimization of dsRNA Length and Target Position for Enhanced RNA Interference Efficiency Targeting the Chitinase Gene MsChi2 in Mythimna separata
Source: Insects. 2026 Sep 4;17(9):931. doi: 10.3390/insects17090931 (PMC13607492; doi:10.3390/insects17090931)
Supplement: Supplementary file 1 [file insects-17-00931-s001.zip › insects-4520967-supplementary.pdf]

Supplementary Table S1 Primer names and sequences used in this study

Primer names	Primer sequences
dsRNA60-F	5'-TAATACGACTCACTATAGGGTCCATTGGTGGCTGGACCTT-3'
dsRNA60-R	5'-TAATACGACTCACTATAGGGTCGCTTTGTCTTGTCGGTGA-3'
dsRNA200-F	5'-TAATACGACTCACTATAGGGAACGTTTTCACTGGTGTAGC-3'
dsRNA200-R	5'-TAATACGACTCACTATAGGGTTCTCGATCGTTGACCTCGC-3'
dsRNA500-F	5'-TAATACGACTCACTATAGGGAGGGTGGTCGTTATCAGATG-3'
dsRNA500-R	5'-TAATACGACTCACTATAGGGAGGAAGGATCTTCAGGTCTG-3'
dsRNA1000-F	5'-TAATACGACTCACTATAGGGACCGAAATAGTCGTTGCCGA-3'
dsRNA1000-R	5'-TAATACGACTCACTATAGGGTTGCCATCCTTGTAGCCGTT -3'
dsRNA200-aF	5'-TAATACGACTCACTATAGGGGTCACGTGGAACGTCTGGTC-3'
dsRNA200-aR	5'-TAATACGACTCACTATAGGGTCGGCAACGACTATTTCGGT-3'
dsRNA200-bF	5'-TAATACGACTCACTATAGGGAGGAAGTTCCCAGTTGACCG-3'
dsRNA200-bR	5'-TAATACGACTCACTATAGGGGGTTTCTGAAGAGCAGCCCA-3'
dsRNA200-cF	5'-TAATACGACTCACTATAGGGTGGAAATTCTTCGATGGTGT-3'
dsRNA200-cR	5'-TAATACGACTCACTATAGGGGGACCAAGCACCCTTGAAGT-3'
dsRNA200-dF	5'-TAATACGACTCACTATAGGGATTCCTGTTGGCTCAGGGAG-3'
dsRNA200-dR	5'-TAATACGACTCACTATAGGGCCCACTTTCCTTGAGCAATT-3'
MsActin-F	5'-CTGAATGGGCGGCTCAAATT-3'
MsActin-R	5'-GGAATCGACAATGTTCCGCA-3'

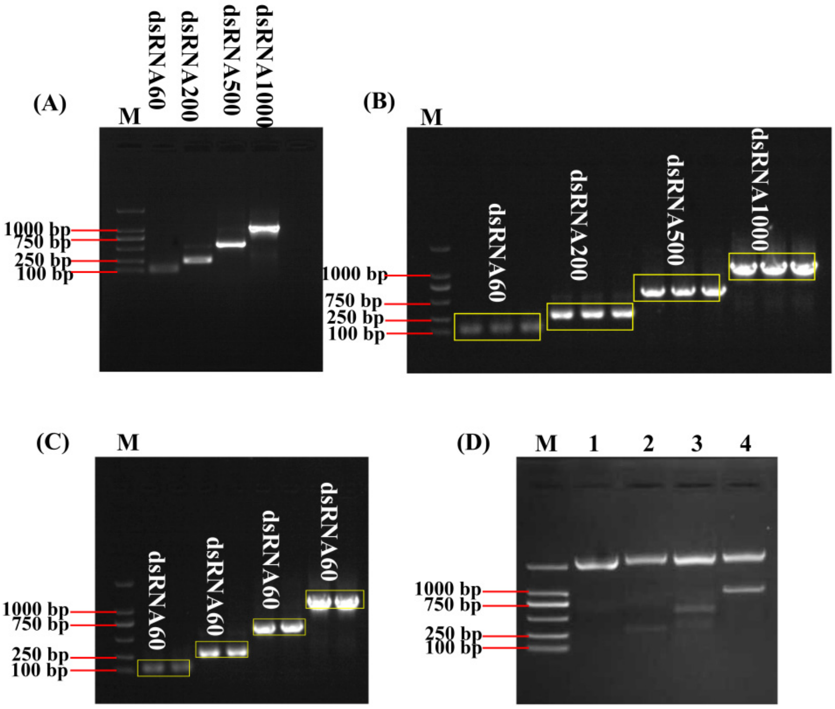

Supplementary Figure S1 Amplification of *MsChi2* fragments of different lengths and identification of recombinant plasmids. (A) Agarose gel electrophoresis of PCR products of target gene fragments; (B) Colony PCR identification; (C) Recombinant plasmid PCR identification; (D) Double digestion with *Hin*dIII and BamHI, M: Trans2K Marker.

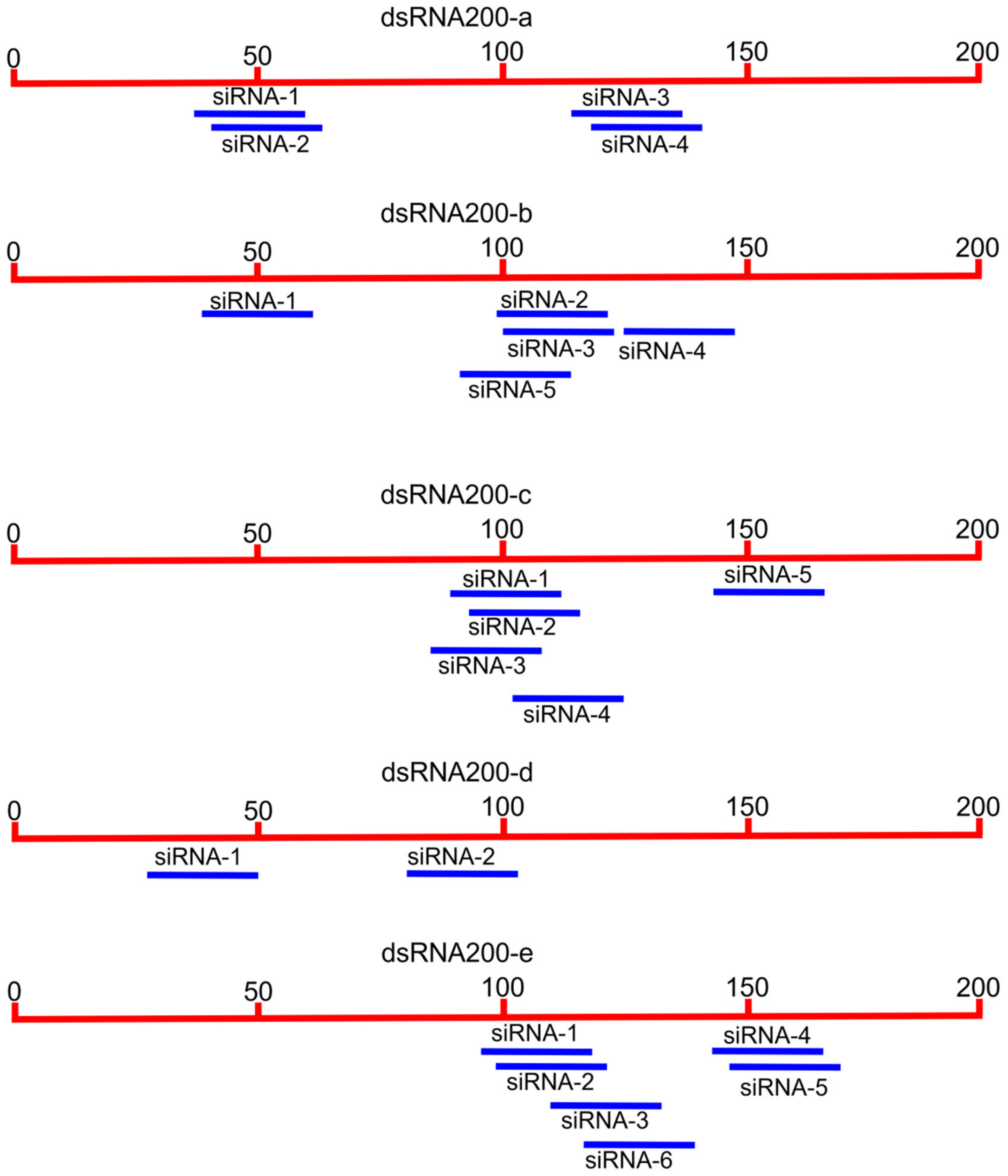

Supplementary Figure S2 Prediction of the relative locations and numbers of effective siRNA candidates within the five 200 bp dsRNA fragments using DNAMAN software. The five fragments, dsRNA200-a through dsRNA200-e, correspond to nucleotide positions 181-380, 499-698, 895-1176, 1260-1459, and 1358-1557 of the *MsChi2* coding sequence, respectively. The predicted effective siRNA sites are indicated by blue vertical bars, and the total number of effective siRNAs for each fragment is labeled as siRNA1, siRNA2, siRNA3, and so on.
